# Clinical retrospective analysis of metformin on TSH levels and nodule volume in patients with type 2 diabetes mellitus combined with thyroid nodules

**DOI:** 10.1097/MD.0000000000047392

**Published:** 2026-02-06

**Authors:** Zheng Ma, Yan Zhang, Liuyun Yuan

**Affiliations:** a Pharmaceutical Department, Cangzhou Central Hospital, Cangzhou, Hebei, China; b Endocrine Diabetes Department, Cangzhou Central Hospital, Cangzhou, Hebei, China.

**Keywords:** insulin resistance, metformin, nodule volume reduction, thyroid nodules, thyroid-stimulating hormone, type 2 diabetes mellitus

## Abstract

Thyroid nodule (TN) prevalence is rising globally, with particularly high rates among patients with type 2 diabetes mellitus, where insulin resistance and hyperinsulinemia may promote nodule formation via the thyroid-stimulating hormone (TSH)/insulin-like growth factor-1 axis. Metformin, the standard first-line therapy for T2DM, may exert additional effects on thyroid structure beyond glycemic control. This single-center retrospective cohort study enrolled 80 patients with T2DM and benign TNs, assigned to a metformin group (n = 40) or control group (n = 40), and followed for 12 months. Changes in TSH and nodule volume were assessed using multivariable regression, propensity score matching, and sensitivity and subgroup analyses to address confounding. After 12 months, metformin treatment was associated with a significant reduction in TSH (adjusted β = −0.32, 95% confidence interval: −0.57 to −0.06, *P* = .017) and nodule volume (adjusted β = −8.5, 95% confidence interval: −15.9 to −1.1, *P* = .024). These effects were consistent in sensitivity analyses, with stronger associations observed in men, patients with body mass index < 28 kg/m², and those with glycated hemoglobin < 8%. In conclusion, metformin significantly lowers TSH levels and reduces nodule size in T2DM patients with benign TNs, supporting its potential clinical utility. Confirmation through large, multicenter, long-term prospective trials is warranted.

## 1. Introduction

Thyroid nodules (TNs) are among the most frequently encountered structural abnormalities of the endocrine system, and their prevalence has shown a marked upward trend worldwide in recent years. In certain populations, particularly with the widespread application of high-resolution ultrasonography, the detection rate has been reported to reach 20 to 50%.^[1]^ Extensive population-based studies suggest that being female, advancing age, obesity, and the presence of metabolic syndrome constitute important risk factors for TN development. The increasing burden associated with these nodules is progressively emerging as a significant issue for both individual health management and public health systems.^[2,3]^ Against this background, the coexistence of TNs in individuals with type 2 diabetes mellitus (T2DM) has become a matter of growing clinical concern. Multiple investigations have demonstrated that metabolic syndrome and its key components, such as excess adiposity, dyslipidemia, and insulin resistance, exert a strong influence on the occurrence of TNs. Moreover, patients with T2DM are more likely not only to exhibit higher nodule detection rates but also to display a more heterogeneous and complicated thyroid morphology.^[4]^

Insulin resistance and compensatory hyperinsulinemia are considered central mechanisms linking T2DM with TN development. Excess insulin and insulin-like growth factor-1 (IGF-1) may potentiate thyroid-stimulating hormone (TSH)-mediated stimulation of thyroid follicular cell proliferation and angiogenesis, thereby promoting nodule growth. In addition, obesity-related chronic low-grade inflammation and alterations in the IGF-1/IGFBP-3 axis further contribute to nodule formation. These findings collectively support the concept that metabolic dysregulation in T2DM promotes nodular thyroid changes through an insulin resistance-IGF-1/TSH axis.^[5,6]^ Taken together, these findings suggest that metabolic derangements in T2DM patients may underlie TN development through an “insulin resistance-IGF-1/TSH axis.”

In the management of T2DM, metformin remains the globally recognized first-line therapeutic agent. Beyond its well-established role in glycemic regulation – achieved primarily through inhibition of hepatic gluconeogenesis and enhancement of insulin sensitivity – it also exerts additional benefits, including modest weight reduction, anti-inflammatory activity, and improvement of the systemic metabolic milieu. Emerging evidence over the past decade has highlighted potential advantages of metformin in the context of thyroid disorders. A systematic review and meta-analysis demonstrated that in patients with concomitant insulin resistance, metformin therapy significantly lowered serum TSH concentrations, improved the homeostasis model assessment of insulin resistance (HOMA-IR) index, and was associated with a reduction in TN size.^[7]^ More recently, a 2023 systematic review reported that the TSH-lowering effect of metformin was more pronounced among individuals with hypothyroidism or those receiving thyroid hormone replacement, whereas its influence was limited in euthyroid populations. These findings suggest that the impact of metformin on thyroid function may be modulated by baseline thyroidal status and underlying metabolic conditions.^[8]^

At the molecular level, metformin appears to influence energy metabolism and cellular proliferation primarily through activation of AMP-activated protein kinase (AMPK) and suppression of the mammalian target of rapamycin (mTOR) signaling cascade. These actions may attenuate the pro-proliferative effects of IGF-1 on thyroid follicular cells and, in parallel, reduce systemic inflammation and oxidative stress, thereby exerting a potential inhibitory impact on nodule progression.^[9]^ Moreover, epidemiological and clinical studies have indicated that long-term metformin exposure in patients with T2DM may be associated with a lower incidence of thyroid cancer, although results remain heterogeneous across investigations.^[10,11]^ Collectively, these findings provide biologically plausible support, together with preliminary clinical data, for a potential role of metformin in modulating TSH levels and structural outcomes in TNs.

Nevertheless, evidence regarding the precise efficacy of metformin in patients with T2DM coexisting with benign TNs remains limited. Many available studies are constrained by small cohorts, short follow-up durations, or a lack of clear distinction for this specific subgroup of patients. Furthermore, systematic investigations are lacking on how baseline metabolic parameters (such as glycated hemoglobin [HbA1c], body mass index [BMI], or HOMA-IR), different TSH strata, or variations in metformin dosage and treatment adherence may influence therapeutic outcomes. By comparison, other glucose-lowering agents, including GLP-1 receptor agonists (GLP-1RA) and SGLT2 inhibitors (SGLT2i), have shown consistent benefits in metabolic regulation; however, direct evidence for their impact on TNs is still scarce. Given its dual role in improving metabolic status and potentially exerting thyroid-protective effects, metformin warrants more rigorous and targeted evaluation in T2DM populations with benign TNs.

Building on this rationale, the present study employed a single-center retrospective cohort design to evaluate the clinical impact of metformin in patients with T2DM and benign TNs. Differences in TSH change (ΔTSH) and nodule volume change (%Δvolume) were compared between the metformin and control groups. To enhance robustness, analyses incorporated propensity score matching, multivariable regression, sensitivity testing, and subgroup as well as interaction assessments. The overarching objective was to clarify the therapeutic value of metformin in this population, address existing knowledge gaps, and provide evidence-based support for the design of future prospective investigations. Despite growing interest in the metabolic determinants of TNs, most existing studies have focused on either general populations, individuals with insulin resistance, or unselected diabetic cohorts. Evidence specifically targeting patients with T2DM who concurrently harbor benign TNs remains scarce. Many available studies lacked rigorous adjustment for confounding factors, had small sample sizes, or did not differentiate between benign and malignant nodules or baseline metabolic strata. Accordingly, the extent to which metformin influences TSH dynamics and structural nodule outcomes in this distinct clinical subgroup is still unclear. These knowledge gaps highlight the need for more focused investigations, such as the present study, which aims to provide population-specific evidence using comprehensive bias-reduction strategies.

## 2. Methodology

### 2.1. Study design and population

This study was approved by the Ethics Committee of Cangzhou Central Hospital. This single-center retrospective cohort study enrolled 80 patients with T2DM and concomitant TNs who were admitted to our hospital between June 2022 and June 2024. All participants fulfilled the World Health Organization (1999) diagnostic criteria for diabetes mellitus and had HbA1c levels ranging from 7 to 10% at baseline. The presence of TNs was confirmed by ultrasonography and, when indicated, fine-needle aspiration cytology. Each nodule was independently assessed by 2 experienced ultrasonographers and verified to be benign with a maximum diameter of <4 cm.

#### 2.1.1. Inclusion criteria

Established diagnosis of T2DM with coexisting benign TNs; nodule diameter < 4 cm; availability of a complete medication history; and no contraindications to the medications evaluated in this study.

#### 2.1.2. Exclusion criteria

Presence of acute diabetic complications, such as ketoacidosis or hyperosmolar hyperglycemic state; pregnancy or coexisting conditions including malignancy, active infection, psychiatric illness, severe gastrointestinal disease, or advanced cardiac, hepatic, or renal dysfunction; prior history of thyroid disease or use of thyroid-related medications; current treatment with drugs known to influence TSH levels (e.g., L-T4, glucocorticoids, dopamine agonists, beta-blockers, retinoic acid, carbamazepine, or metyrapone); diagnosis of hyperthyroidism or hypothyroidism; and ultrasound findings with potential confounding factors, such as retrosternal thyroid cysts or thyroid volume >40 mL.

A total of 80 patients were ultimately included and allocated to either the metformin group (n = 40) or the control group (n = 40) based on their glucose-lowering treatment regimen.

### 2.2. Interventions

All participants received standardized dietary and exercise guidance along with diabetes-related health education.

#### 2.2.1. Metformin group

Patients were treated with oral metformin, initiated at 0.25 g twice daily. The dose was increased by 0.50 g each week as tolerated, up to a maximum daily dose of 2.55 g, administered in 2 divided doses.

#### 2.2.2. Control group

Patients received non-metformin glucose-lowering therapies (e.g., DPP-4 inhibitors, SGLT2i, or GLP-1RA), with insulin added if clinically indicated.

Medication adherence was evaluated through prescription records and patient interviews, and the medication possession ratio was calculated. All patients maintained their respective regimens for 12 months.

### 2.3. Detection indicators

#### 2.3.1. TN assessment

Nodule length, width, and thickness were measured using a high-resolution ultrasound system (e.g., GE Logiq E9) by the same senior sonographer. Nodule volume was calculated with the formula: volume = length × width × thickness × 0.52 (mL). Maximum nodule diameter, number of nodules, and Thyroid Imaging Reporting and Data System (TI-RADS) grade were documented. To reduce measurement bias, a subset of samples was independently reevaluated by 2 physicians.

#### 2.3.2. Thyroid function

Serum free triiodothyronine (FT3), free thyroxine (FT4), and TSH were quantified using an automated chemiluminescent analyzer (Cobas e602, Roche Diagnostics Ltd., Rotkreuz, Switzerland). The reference ranges were 3.28 to 6.47 pmol/L, 7.9 to 18.4 pmol/L, and 0.34 to 5.60 mIU/L, respectively.

#### 2.3.3. Metabolic parameters

Fasting blood glucose (FBG) and 2-hour postprandial blood glucose were measured using the glucose oxidase method; HbA1c was determined by high-performance liquid chromatography; and fasting insulin (FINS) was assessed by chemiluminescence. The HOMA-IR was calculated as: FBG (mmol/L) × FINS (mIU/L)/22.5.

#### 2.3.4. IGF-1 level

Serum IGF-1 was determined using chemiluminescence.

### 2.4. Endpoint indicators

#### 2.4.1. Primary endpoint

Change in serum TSH after 12 months of follow-up (ΔTSH = follow-up value − baseline value).

#### 2.4.2. Secondary endpoints

Relative change in TN volume (%Δvolume), changes in FT3, FT4, IGF-1, and HbA1c, as well as the incidence of TI-RADS grade progression and the requirement for additional puncture or procedures. TI-RADS progression and additional interventions were considered exploratory outcomes.

### 2.5. Statistical analysis

All analyses were conducted using SPSS version 19.0 (IBM Corp., Armonk).

#### 2.5.1. Descriptive statistics

Data normality was assessed with the Shapiro–Wilk test. Variables with a normal distribution were expressed as mean ± standard deviation (Mean ± SD) and compared between groups using the independent-samples *t*-test. Non-normally distributed variables were presented as median (interquartile range) and analyzed using the Mann–Whitney *U* test. Categorical variables were reported as counts and percentages, with group comparisons performed using the χ² test or Fisher's exact test as appropriate.

#### 2.5.2. Propensity score analysis

1:1 nearest-neighbor matching (caliper width of 0.2 SD) was applied, supplemented by inverse probability of treatment weighting. A standardized mean difference <0.1 after matching was considered indicative of adequate balance.

#### 2.5.3. Regression modeling

Multivariable linear regression was performed to evaluate the association between metformin use and ΔTSH, adjusting for clinically relevant covariates and those showing baseline imbalance.

#### 2.5.4. Sensitivity and subgroup analyses

Sensitivity analyses were conducted by excluding patients on TSH-influencing drugs, restricting to individuals with normal FT3/FT4, modifying adherence thresholds, and performing intention-to-treat analyses. Subgroup analyses were stratified by sex, BMI, HOMA-IR, baseline TSH, maximum nodule diameter, and HbA1c level.

A 2-sided *P* < .05 was considered statistically significant.

## 3. Results

### 3.1. Comparison of baseline characteristics

A total of 80 patients with T2DM and coexisting TNs were included, comprising 40 in the metformin group and 40 in the control group. Baseline characteristics, including age, sex, BMI, diabetes duration, smoking status, alcohol consumption, HbA1c, FBG, postprandial blood glucose, FINS, HOMA-IR, TSH, FT3, FT4, IGF-1, and TN features (maximum diameter, volume, number, and TI-RADS grade), showed no significant differences between groups (all *P* > .05). Similarly, the use of glucose-lowering medications (insulin, GLP-1RA, SGLT2i, DPP-4 inhibitors) and statins did not differ significantly between the 2 groups (Table 1).

**Table 1:** Baseline characteristics comparison.

Variables	Overall	Metformin group	Control group	SMD	*P*-value (test)
Sample size	80	40	40	–	–
Age (yr)	56.0 ± 9.1	55.2 ± 9.1	56.8 ± 9.2	−0.17	.441
Female, n (%)	48 (60.0%)	28 (70.0%)	20 (50.0%)	0.42	.11
BMI (kg/m²)	26.8 ± 3.4	26.5 ± 2.8	27.1 ± 4.0	−0.17	.457
Duration of diabetes (yr)	6.47 (3.17–10.25)	6.00 (3.40–9.70)	7.28 (2.89–12.75)	−0.18	.881
Smoking, n (%)	17 (21.2%)	7 (17.5%)	10 (25.0%)	−0.18	.585
Alcohol consumption, n (%)	18 (22.5%)	12 (30.0%)	6 (15.0%)	0.37	.181
HbA1c (%)	7.8 ± 1.0	7.8 ± 1.1	7.8 ± 0.9	−0.01	.977
FBG (mmol/L)	8.6 ± 1.8	8.6 ± 1.8	8.6 ± 1.9	0.03	.884
PBG (mmol/L)	11.9 ± 3.1	11.9 ± 3.2	12.0 ± 3.0	−0.03	.906
FINS (μIU/mL)	9.23 (6.55–13.08)	8.89 (6.92–13.21)	9.34 (5.68–12.72)	0.13	.725
HOMA-IR	3.67 (2.34–4.66)	3.64 (2.34–4.78)	3.67 (2.44–4.62)	0.13	.516
TSH (mIU/L)	2.33 (1.79–2.88)	2.14 (1.83–2.62)	2.52 (1.78–3.07)	−0.25	.22
FT4 (pmol/L)	15.1 ± 2.2	15.0 ± 2.4	15.2 ± 2.1	−0.06	.693
FT3 (pmol/L)	4.5 ± 0.5	4.6 ± 0.5	4.5 ± 0.5	0.18	.374
IGF-1 (ng/mL)	135.2 ± 37.4	138.0 ± 34.6	132.4 ± 40.2	0.15	.505
Maximum nodule diameter (mm)	13.44 (11.76–14.95)	13.39 (11.46–14.44)	13.46 (12.39–15.10)	−0.12	.491
Nodule volume (mL)	0.50 (0.31–0.72)	0.48 (0.30–0.59)	0.51 (0.36–0.81)	−0.34	.292
Number of nodules (≥2), n (%)	30 (37.5%)	14 (35.0%)	16 (40.0%)	−0.10	.817
TI-RADS ≥ 4, n (%)	32 (40.0%)	18 (45.0%)	14 (35.0%)	0.21	.494
Antidiabetic medication: Insulin, n (%)	29 (36.2%)	13 (32.5%)	16 (40.0%)	−0.16	.642
Antidiabetic medication: GLP-1RA, n (%)	14 (17.5%)	8 (20.0%)	6 (15.0%)	0.13	.769
Antidiabetic medication: SGLT2i, n (%)	16 (20.0%)	7 (17.5%)	9 (22.5%)	−0.13	.78
Antidiabetic medication: DPP-4i, n (%)	20 (25.0%)	11 (27.5%)	9 (22.5%)	0.12	.796
Statin use, n (%)	41 (51.2%)	22 (55.0%)	19 (47.5%)	0.15	.655
Iodine intake (iodized salt), n (%)	64 (80.0%)	29 (72.5%)	35 (87.5%)	−0.38	.162

BMI = body mass index, DPP-4i = DPP-4 inhibitors, FBG = fasting blood glucose, FINS = fasting insulin, FT3 = free triiodothyronine, FT4 = free thyroxine, GLP-1RA = GLP-1 receptor agonists, HbA1c = glycated haemoglobin, HOMA-IR = homeostasis model assessment of insulin resistance, IGF-1 = insulin-like growth factor-1, PBG = postprandial blood glucose, SGLT2i = SGLT2 inhibitors, SMD = standardized mean difference, TI-RADS = Thyroid Imaging Reporting and Data System, TSH = thyroid-stimulating hormone.

### 3.2. Comparison of treatment outcomes

After 12 months of follow-up, TSH levels decreased significantly in the metformin group (ΔTSH: −0.27 ± 0.61 mIU/L), whereas a slight increase was observed in the control group (ΔTSH: 0.08 ± 0.50 mIU/L). The between-group difference was −0.34 (95% confidence interval [CI]: −0.59 to −0.09), with a multivariable-adjusted effect of −0.32 (95% CI: −0.57 to −0.06, *P* = .017).

TN volume declined more markedly in the metformin group compared with the control group (−10.64 ± 13.53% vs −0.70 ± 18.96%), and the difference remained significant after adjustment (β = −8.5, 95% CI: −15.9 to −1.1, *P* = .024).

No significant between-group differences were detected in FT3, FT4, IGF-1, or HbA1c levels (all *P* > .05). Likewise, the incidence of TI-RADS grade progression and the requirement for new puncture or additional procedures did not differ significantly between groups (Table 2).

**Table 2:** Comparison of main outcome indicators.

Outcome variables	Metformin group (mean ± SD or *n*/%)	Control group (mean ± SD or *n*/%)	Crude effect (difference/ratio/β)	95% CI	Adjusted effect (β or OR)	95% CI (adjusted)	*P*-value (adjusted)
ΔTSH (mIU/L)	−0.27 ± 0.61	0.08 ± 0.50	−0.34	−0.59 to −0.09	−0.32	−0.57 to −0.06	.017
%Δ Nodule volume (%)	−10.64 ± 13.53	−0.70 ± 18.96	−9.9	−17.2 to −2.6	−8.5	−15.9 to −1.1	.024
ΔFT4 (pmol/L)	0.05 ± 1.49	0.46 ± 1.45	−0.40	−1.06 to 0.25	−0.38	−1.07 to 0.30	.270
ΔFT3 (pmol/L)	0.02 ± 0.41	−0.12 ± 0.39	0.14	−0.04 to 0.31	0.10	−0.08 to 0.28	.261
ΔIGF-1 (ng/mL)	−5.64 ± 16.49	−5.70 ± 20.77	0.1	−8.3 to 8.4	−1.7	−10.2 to 6.8	.692
ΔHbA1c (%)	−0.54 ± 0.83	−0.28 ± 0.85	−0.26	−0.64 to 0.11	−0.28	−0.67 to 0.11	.156
TI-RADS upgrading (yes/no)	3 (7.5%)	3 (7.5%)	OR = 1.00	0.19 to 5.28	–	–	–
New biopsy/surgery (yes/no)	3 (7.5%)	2 (5.0%)	OR = 1.54	0.24 to 9.75	–	–	–

CI = confidence interval, FT3 = free triiodothyronine, FT4 = free thyroxine, HbA1c = glycated haemoglobin, IGF-1 = insulin-like growth factor-1, OR = odds ratio, TI-RADS = Thyroid Imaging Reporting and Data System, TSH = thyroid-stimulating hormone.

### 3.3. Propensity score and sensitivity analysis

After propensity score matching and weighting, baseline covariates were well balanced (weighted standardized mean difference < 0.1) (Table 3).

**Table 3:** Propensity score matching and weighting results (covariate balance).

Covariate	SMD before matching	SMD after matching	SMD after weighting
Age	−0.17	0.04	0.03
Sex (female)	0.42	0.08	0.06
BMI	−0.17	0.04	0.03
Duration of diabetes	−0.18	0.04	0.03
HbA1c	−0.01	0.00	0.00
HOMA-IR	0.13	0.03	0.02
TSH	−0.25	0.06	0.05
FT4	−0.06	0.01	0.01
FT3	0.18	0.05	0.03
IGF-1	0.15	0.04	0.03
Maximum nodule diameter	−0.12	0.03	0.02
Nodule volume	−0.34	0.08	0.06
Antidiabetic medication categories (indicator variables)	0.05	0.01	0.01
Statin use	0.15	0.04	0.03

BMI = body mass index, FT3 = free triiodothyronine, FT4 = free thyroxine, HbA1c = glycated haemoglobin, HOMA-IR = homeostasis model assessment of insulin resistance, IGF-1 = insulin-like growth factor-1, SMD = standardized mean difference, TSH = thyroid-stimulating hormone.

Sensitivity analyses demonstrated that the TSH-lowering effect of metformin was consistent across multiple scenarios, with β values ranging from −0.28 to −0.32 (all *P* < .05). These results were robust after excluding patients on TSH-influencing medications, restricting analyses to those with normal FT3/FT4, modifying adherence thresholds, and applying an intention-to-treat approach. In contrast, this effect was not evident in the negative control analysis using DPP-4 inhibitor users (Table 4).

**Table 4:** Results of sensitivity analysis.

Sensitivity analysis setting	Effect estimate (β/OR/HR)	95% CI	*P*-value
Remove those who affect TSH medications	−0.32	−0.57 to −0.06	.017
Limit those with normal FT3/FT4	−0.30	−0.55 to −0.06	.017
Varying MPR threshold (0.7/0.9)	−0.29	−0.52 to −0.05	.017
Intentional exposure (ITT)	−0.28	−0.51 to −0.05	.018
Negative control for control drug (e.g., DPP-4i)	−0.02	−0.18 to 0.14	.80

CI = confidence interval, DPP-4i = DPP-4 inhibitors, FT3 = free triiodothyronine, FT4 = free thyroxine, HR = hazards ratio, ITT = intention-to-treat, MPR = medication possession ratio, OR = odds ratio, TSH = thyroid-stimulating hormone.

### 3.4. *Dose–response and subgroup analysis*

Dose-stratified analysis indicated that patients receiving medium-dose metformin (~1500 mg/day) exhibited the greatest reduction in TSH (β = −0.29, 95% CI: −0.59 to 0.01, *P* = .058); however, none of the between-dose group comparisons reached statistical significance. Similarly, stepwise dose analysis (per additional 500 mg) showed no correlation with TSH changes (Table 5).

**Table 5:** Dose–response analysis.

Dose stratification/continuous variable	N (exposed)	Effect on primary outcome (β: ΔTSH)	95% CI	*P*-value
Low dose	12	−0.24	−0.56 to 0.08	.142
Medium dose	15	−0.29	−0.59 to 0.01	.058
High dose	13	−0.26	−0.64 to 0.12	.18
Continuous dose (per 500 mg)	–	0.020	−0.238 to 0.264	.876

TSH = thyroid-stimulating hormone.

Subgroup analyses revealed a significant TSH-lowering effect of metformin in male patients (β = −0.55, *P* = .011), individuals with BMI < 28 kg/m² (β = −0.46, *P* = .006), those with a maximum nodule diameter ≥ 10 mm (β = −0.32, *P* = .016), and patients with HbA1c < 8% (β = −0.66, *P* < .001) (Table 6). Interaction testing further demonstrated a significant effect modification by HbA1c level on the association between metformin and TSH (interaction *P* = .003).

**Table 6:** Results of subgroup analysis.

Subgroup	Level	N (exposed/control)	Effect (β or OR)	95% CI	*P*-value	Interaction *P*-value
Sex	Male	12/20	−0.55	−0.96 to −0.14	.011	.225
	Female	28/20	−0.21	−0.58 to 0.15	.241	
BMI	<28	28/27	−0.46	−0.78 to −0.14	.006	.151
	≥28	12/13	0.00	−0.54 to 0.54	.991	
HOMA-IR	Low (<median)	20/20	−0.30	−0.73 to 0.12	.157	.775
	High (≥median)	20/20	−0.22	−0.57 to 0.12	.191	
Baseline TSH	Q1	10/10	−0.39	−1.02 to 0.24	.203	.715
	Q4	6/14	−0.55	−1.13 to 0.04	.064	
Maximum nodule diameter	<10 mm	2/2	−0.05	NaN to NaN	NaN	–
	≥10 mm	38/38	−0.32	−0.58 to −0.06	.016	
HbA1c	<8%	26/23	−0.66	−0.92 to −0.39	.000	.003
	≥8%	14/17	0.23	−0.30 to 0.76	.377	

BMI = body mass index, CI = confidence interval, HbA1c = glycated hemoglobin, HOMA-IR = homeostasis model assessment of insulin resistance, NaN = not a number, OR = odds ratio, TSH = thyroid-stimulating hormone.

## 4. Discussion

The findings of this study demonstrated that metformin significantly lowered TSH levels and reduced nodule size in patients with T2DM and benign TNs. These results offer additional evidence supporting the potential therapeutic value of metformin in this population and are consistent with trends reported in prior studies.^[12,13]^

With respect to TSH, this study identified a significant reduction in the metformin group after 12 months of follow-up. Prior systematic reviews and meta-analyses have similarly reported that metformin can lower TSH levels in insulin-resistant populations, accompanied by improvements in HOMA-IR.^[13]^ Another investigation indicated that the TSH-lowering effect of metformin was more evident in patients with hypothyroidism or those receiving replacement therapy, whereas its influence was limited in individuals with normal thyroid function and metabolic profiles.^[14]^ These findings suggest that the thyroidal effect of metformin may depend on baseline thyroid function and metabolic status, which could account for the more pronounced benefit observed in T2DM patients with metabolic abnormalities.

Regarding nodule volume, this study provides clear evidence that metformin significantly reduces the size of benign TNs, consistent with prior research. The IGF-1/IGFBP-3 ratio has been identified as an important predictor of nodule progression.^[15]^ Bai and Chen^[16]^ reported that metformin therapy not only reduced nodule volume but also decreased oxidative stress levels. Similarly, a randomized controlled trial by Cho et al^[17]^ demonstrated reductions in thyroid volume alongside suppression of TSH with metformin treatment. Collectively, our results reinforce and extend these findings, highlighting the potential clinical relevance of metformin in improving structural outcomes.

From a mechanistic perspective, metformin may regulate energy metabolism and cellular proliferation through activation of AMPK and inhibition of the mTOR signaling pathway.^[18]^ Additionally, its anti-inflammatory and antioxidant properties may help optimize the thyroid microenvironment, thereby slowing or even reversing the development and progression of nodules. Experimental studies by Han et al^[19]^ further demonstrated that metformin reduces pro-inflammatory cytokine expression and lowers ROS production, conferring protective effects on endocrine tissues. These mechanistic insights provide a plausible explanation for the clinical outcomes observed in this study.

Epidemiological evidence also indicates that metformin may be linked to a lower risk of thyroid cancer. A systematic review by Celescuekci et al^[20]^ reported that long-term metformin use was associated with a ~30% reduction in thyroid cancer risk. Another meta-analysis suggested that metformin could have a favorable impact on the prognosis of patients with thyroid cancer.^[21]^ Similarly, Zhang et al^[22]^ found that metformin use was correlated with reduced tumor burden in individuals with T2DM and thyroid cancer. Although the present study focused on benign nodules, these findings collectively highlight the potential multifaceted role of metformin across the spectrum of thyroid diseases.

From a clinical standpoint, managing patients with T2DM and concomitant TNs is challenging, as targeted interventions for nodule-related structural outcomes remain limited. If metformin can simultaneously reduce TSH levels and shrink nodule size while improving glycemic control, it may offer a comprehensive “one-drug-fits-all” therapeutic approach for this population.^[23]^ Moreover, the direct evidence supporting metformin’s effect on TNs is currently stronger than that for newer glucose-lowering agents such as GLP-1RA and SGLT2i, providing additional justification for its application in this specific setting.

This study offers several innovative aspects: it specifically targeted patients with T2DM and benign TNs, employed propensity score matching and sensitivity analyses to minimize confounding, and examined the heterogeneity of drug effects through subgroup and interaction analyses across baseline characteristics. The findings indicate that metformin exerts a more pronounced TSH-lowering effect in men, in patients with lower HbA1c, and in those with lower BMI, consistent with prior evidence suggesting that “better glycemic control is associated with a reduced risk of nodules.” Additional exploratory analyses were performed to evaluate whether baseline nodule characteristics influenced the volume-reducing effect of metformin. When stratified by single versus multiple nodules and solid versus mixed cystic–solids nodules, no statistically significant interaction was observed (all *P* for interaction > .10). The direction and magnitude of nodule volume reduction remained consistent across nodule types, suggesting that the structural response to metformin is not substantially modified by baseline morphological classification.

However, this study has several limitations. First, as a single-center retrospective design, causal inference cannot be fully established, and selection bias may still be present. Second, nodule volume was assessed by ultrasound, which could affect measurement accuracy despite the use of dual independent evaluations to minimize error. Third, the 12-month follow-up was relatively short and did not capture long-term outcomes such as malignant transformation or the need for surgery. Fourth, this study did not clarify the potential dose–response relationship between metformin dosage and nodule changes, which warrants further investigation. Finally, the sample size was modest, limiting the statistical power of some subgroup analyses.

Looking ahead, large-scale multicenter randomized controlled trials with extended follow-up are required to confirm the long-term efficacy and safety of metformin in patients with T2DM and benign TNs.^[23]^ In addition, defining the criteria for individualized patient benefit should be further investigated and integrated with mechanistic studies to clarify the molecular pathways underlying metformin’s effects. Future research incorporating novel glucose-lowering agents alongside metformin would also enable a more comprehensive evaluation of their impact on structural thyroid outcomes. Compared with previous studies, this work provides several unique contributions. First, it focuses specifically on patients with both T2DM and benign TNs, a subgroup underrepresented in prior research. Second, by incorporating propensity score matching, inverse probability of treatment weighting, multivariable modeling, and multiple sensitivity analyses, our study offers a more robust estimation of metformin’s structural thyroid effects than earlier observational studies. Third, the identification of effect heterogeneity – particularly the stronger TSH reduction in men, patients with lower BMI, and those with HbA1c < 8% – adds clinically relevant insight into which patient subgroups may derive the greatest benefit. These findings refine existing evidence and help establish a more precise therapeutic framework for future clinical practice.

In conclusion, this study indicates that metformin may lower TSH levels and significantly reduce the size of benign TNs in patients with T2DM, potentially through improving insulin resistance, modulating the IGF-1/TSH axis, activating the AMPK/mTOR pathway, and exerting anti-inflammatory and antioxidant effects. These findings align with prior mechanistic and clinical evidence and provide preliminary support for the broader application of metformin in individuals with diabetes and TNs. Future large-scale randomized controlled trials are warranted to establish long-term efficacy and safety, and to define criteria for identifying patients most likely to benefit, thereby strengthening the clinical basis for treatment.

## Author contributions

**Conceptualization:** Zheng Ma, Yan Zhang, Liuyun Yuan.

**Data curation:** Zheng Ma, Yan Zhang, Liuyun Yuan.

**Formal analysis:** Zheng Ma, Yan Zhang, Liuyun Yuan.

**Funding acquisition:** Zheng Ma, Yan Zhang, Liuyun Yuan.

**Investigation:** Zheng Ma, Yan Zhang.

**Writing – original draft:** Zheng Ma, Yan Zhang, Liuyun Yuan.

**Writing – review & editing:** Zheng Ma, Yan Zhang, Liuyun Yuan.
